# Untargeted high-resolution plasma metabolomic profiling predicts outcomes in patients with coronary artery disease

**DOI:** 10.1371/journal.pone.0237579

**Published:** 2020-08-18

**Authors:** Anurag Mehta, Chang Liu, Aditi Nayak, Ayman S. Tahhan, Yi-An Ko, Devinder S. Dhindsa, Jeong Hwan Kim, Salim S. Hayek, Laurence S. Sperling, Puja K. Mehta, Yan V. Sun, Karan Uppal, Dean P. Jones, Arshed A. Quyyumi

**Affiliations:** 1 Emory Clinical Cardiovascular Research Institute, Division of Cardiology, Department of Medicine, Emory University School of Medicine, Atlanta, Georgia; 2 Department of Epidemiology, Rollins School of Public Health, Emory University, Atlanta, Georgia; 3 Department of Biostatistics and Bioinformatics, Rollins School of Public Health, Emory University, Atlanta, Georgia; 4 Division of Cardiology, Department of Internal Medicine, University of Michigan Medical School, Ann Arbor, Michigan; 5 Atlanta VA Health Care System, Decatur, Georgia; 6 Division of Pulmonary, Allergy, Critical Care and Sleep Medicine, Department of Medicine, Emory University School of Medicine, Atlanta, Georgia; Queen's University Belfast, UNITED KINGDOM

## Abstract

**Objective:**

Patients with CAD have substantial residual risk of mortality, and whether hitherto unknown small-molecule metabolites and metabolic pathways contribute to this risk is unclear. We sought to determine the predictive value of plasma metabolomic profiling in patients with CAD.

**Approach and results:**

Untargeted high-resolution plasma metabolomic profiling of subjects undergoing coronary angiography was performed using liquid chromatography/mass spectrometry. Metabolic features and pathways associated with mortality were identified in 454 subjects using metabolome-wide association studies and Mummichog, respectively, and validated in 322 subjects. A metabolomic risk score comprising of log-transformed HR estimates of metabolites that associated with mortality and passed LASSO regression was created and its performance validated. In 776 subjects (66.8 years, 64% male, 17% Black), 433 and 357 features associated with mortality (FDR-adjusted q<0.20); and clustered into 21 and 9 metabolic pathways in first and second cohorts, respectively. Six pathways (urea cycle/amino group, tryptophan, aspartate/asparagine, lysine, tyrosine, and carnitine shuttle) were common. A metabolomic risk score comprising of 7 metabolites independently predicted mortality in the second cohort (HR per 1-unit increase 2.14, 95%CI 1.62, 2.83). Adding the score to a model of clinical predictors improved risk discrimination (delta C-statistic 0.039, 95%CI -0.006, 0.086; and Integrated Discrimination Index 0.084, 95%CI 0.030, 0.151) and reclassification (continuous Net Reclassification Index 23.3%, 95%CI 7.9%, 38.2%).

**Conclusions:**

Differential regulation of six metabolic pathways involved in myocardial energetics and systemic inflammation is independently associated with mortality in patients with CAD. A novel risk score consisting of representative metabolites is highly predictive of mortality.

## Introduction

Patients with established coronary artery disease (CAD) are at a high risk of mortality and CAD is the leading cause of death in the United States. [1] The current paradigm of risk assessment among patients with CAD involves ascertainment of high-risk clinical characteristics that are known to portend adverse outcomes. [2] This approach is imperfect and does not provide information regarding pathobiological factors responsible for the increased ‘residual’ disease risk observed among some patients with CAD. [3] Additionally, the precise metabolic pathways underlying this risk of adverse outcomes are not well elucidated. Improved understanding of these pathways may help provide key insights into complex disease mechanisms, personalize risk assessment by identifying novel risk markers that prognosticate outcomes, and potentially discern targets for therapeutic interventions in this high-risk patient population.

The emerging role of metabolomics profiling in this conceptual framework has been recently described in a scientific statement from the American Heart Association (AHA). [4] Metabolomics is the systematic study of small-molecule metabolites across biological systems, and the metabolome constitutes the final downstream product of many regulatory complexes (genome→ transcriptome→ proteome→ metabolome) that are proximal to a disease phenotype. [4] Technical advances in *targeted* metabolomics data mapping to biological pathways has recently provided important insights into the pathobiology of CAD. For example, one study demonstrated a strong association of arginine and its downstream metabolites ornithine and citrulline, key substrates in the nitric oxide synthesis pathway, with major adverse cardiovascular events among patients with CAD. [5] Another recent study showed that significant alteration in metabolism of phospholipids, amino acids, short-chain acylcarnitines, and primary bile acids is associated with CAD severity among patients undergoing coronary angiography. [6]

Nevertheless, there is a paucity of studies evaluating the prognostic utility of *untargeted* metabolomics among patients with CAD. Untargeted metabolomics is an approach that focuses on global detection and relative quantitation of small-molecule metabolites to understand both known and unknown metabolic changes that accompany disease states. [7] Herein, we used untargeted high-resolution plasma metabolomic profiling to identify metabolic pathways associated with mortality among patients with CAD undergoing cardiac catheterization. We have additionally created and internally validated a novel metabolomic risk score to predict mortality risk in our cohort.

## Materials and methods

### Study population

We studied subjects enrolled in the Emory Cardiovascular Biobank–an ongoing prospective registry of patients undergoing cardiac catheterization for evaluation of suspected or known CAD at three Emory Healthcare affiliated hospitals in Atlanta, Georgia, USA. [8] In the current study we evaluated subjects that underwent untargeted high-resolution plasma metabolomic profiling at different time points and were part of two distinct cohorts. Subjects in the first cohort were enrolled in the years 2004 to 2005, underwent plasma metabolomic profiling in 2012, and were sex/race propensity matched 1:1 for the presence or absence of all-cause mortality during follow-up. Subjects in the second cohort were enrolled in the years 2004 to 2011, underwent metabolomic profiling in 2016, and were sex/race propensity matched 1:2 for the presence or absence of all-cause mortality during follow-up.

Participants in both cohorts were interviewed to collect information about demographic characteristics, medical history, and behavioral habits as previously described. [8] The prevalence of hypertension, diabetes, heart failure (HF), peripheral artery disease (PAD), stroke, and prior coronary artery bypass grafting (CABG) was determined by physician diagnosis and/or treatment. [8] Medical records and International Classification of Diseases (ICD)-9 diagnostic codes were reviewed to confirm self-reported medical history. Weight and height were measured at enrollment and body mass index (BMI) was calculated by dividing weight (in kilogram) by height (in meters)-square. Left ventricular ejection fraction (LVEF) was obtained from medical records and estimated glomerular filtration rate (eGFR) was calculated using the Chronic Kidney Disease Epidemiology Collaboration equation. [9] Serum high-sensitivity C-reactive protein (hs-CRP), plasma high-sensitivity cardiac Troponin-I (hs-cTnI), and serum N-terminal prohormone of brain natriuretic peptide (NT-proBNP) levels were measured using the using a sandwich immunoassay (in mg/L, FirstMark Inc., San Diego, CA), Alere NT-proBNP (in pg/mL, Abbott Laboratories, Inc.), and STAT Troponin-I assays (in pg/mL, Abbott Laboratories, Inc.), respectively. All patients were stable at the time of enrollment and those with myocardial infarction, defined using international criteria, [10] were also included if clinically stable.

Subjects with data available regarding age, sex, and race, and prospective follow-up for all-cause mortality were included (n = 494 for first and 440 for second cohort); while those with cardiac transplantation, history of non-ischemic cardiomyopathy, and follow-up duration less than 30 days were excluded (n = 40 and 118, respectively). Thus, 454 subjects in the first cohort and 322 subjects in the second cohort were analyzed. This study complies with the Declaration of Helsinki and was approved by the institutional review board at Emory University (Atlanta, Georgia) under IRB00000343. All subjects provided written informed consent at the time of enrollment.

### High-resolution metabolomic profiling

All subjects underwent an overnight fast before blood collection and untargeted high-resolution metabolomic profiling using liquid-chromatography mass spectrometry (LC/MS) was performed using standardized techniques after thawing each subject’s plasma stored at -80° Celsius. [11–17] Plasma samples were run in a randomized order in batches of 20 and three technical replicates were analyzed for each sample in a sequential manner. Plasma aliquots (65 μL) were treated with 130 μL acetonitrile (2:1 volume/volume) containing 3.5 μL of an internal isotopic standard mix and placed on ice for 30 minutes. [14–17] The internal standard mix consisted of 14 stable isotopic chemicals, [14] which cover a broad range of chemical properties represented in small molecules: [^13^C_6_]-d-glucose, [^15^N]-indole, [2-^15^N]-l-lysine dihydrochloride, [^13^C_5_]-l-glutamic acid, [^13^C_7_]-benzoic acid, [3,4-^13^C_2_]-cholesterol, [^15^N]-l-tyrosine, [trimethyl-^13^C_3_]-caffeine, [^15^N_2_]-uracil, [3,3-^13^C_2_]-cystine, [1,2-^13^C_2_]-palmitic acid, [^15^N,^13^C_5_]-l-methionine, [^15^N]-choline chloride, and 2’-deoxyguanosine-^15^N_2_,^13^C_10_-5’-monophosphate. Samples were analyzed using a Thermo LTQ Velos Orbitrap high-resolution (60,000 mass resolution) mass spectrometer (Thermo Fisher Scientific, San Diego, California) and C18 column chromatography (Higgins Analytical Inc., Targa, 2.1 × 10 cm) in positive ionization mode with a scanning m/z range of 85–2000 over 10 minutes. [11, 18] Elution was obtained with a formic acid/acetonitrile gradient at a flow rate of 0.35 mL/min for the initial 6 minutes and 0.50 mL/min for the remaining 4 minutes. The first 2-minute period consisted of 5% solution A (2% volume/volume formic acid in water), 60% water, and 35% acetonitrile. This was followed by a 4-min linear gradient to 5% solution A, 0% water, and 95% acetonitrile. The final 4-minute period was maintained at 5% solution A and 95% acetonitrile. For quality control and assurance, pooled reference plasma was run before and after each batch of 20 samples. The average Pearson correlation coefficient and coefficient of variation (%) within the quality cohort (QC) samples for the first cohort were 0.96 and 11.3%, respectively. The corresponding values within the QC samples for the second cohort were 0.95 and 10.5%, respectively. In addition, principal component analysis (PCA) of the QC and study samples was performed to evaluate batch-effects (S1 Fig). Although the pairwise correlation between QC samples were high in both cohorts, batch-effects were observed and therefore we included batch as a covariate in the regression models as described below. Ion dissociation mass spectrometry (LC-MS/MS) analysis was performed using the same protocols as LC/MS using high purity N_2_ at normalized collision energy of 35%. The tandem mass spectrometry data were processed using the *xcmsFragments* function in XCMS, [19–21] and the experimental spectra were compared with in-silico fragmentation using MetFrag. [22]

### Data processing

Raw data files were processed into the computable document format (.cdf) format using Xcalibur file converter software (Thermo Fisher, San Diego, California). The adaptive processing software package, apLCMS (available at http://web1.sph.emory.edu/apLCMS/), designed for use with high-resolution mass spectrometry data, was used for noise removal and feature extraction, alignment, and quantification. [23] All metabolic features were identified using a unique combination of m/z and retention time. The mean feature intensity value was used for analysis, which was calculated from the mean of non-zero readings for each feature of every subject. A feature was retained for further analysis if at least 80% of subjects had non-zero intensity reading. After exclusion, the zero mean intensity values were treated as truly zero intensities. All intensity values were log_2_ transformed [log_2_*(m+*1), *m* = feature intensity], mean centered and scaled by standard deviation. A total of 6,781 features entered analysis in the first cohort and 8,714 in the second cohort.

### Follow-up and outcomes

Study subjects were prospectively followed for the primary outcome of all-cause mortality and the secondary outcome of cardiovascular death. Outcome censoring was performed at 3 years and follow-up data was obtained by annual phone contact, electronic medical record review, and data from the social security death index and state records. [8] Cardiovascular death was defined as death attributable to an ischemic cardiovascular cause like fatal MI, stroke or sudden death secondary to a presumed cardiovascular cause in this high-risk population and cardiovascular death events were adjudicated by two cardiologists who were blinded to study data. [24]

### Statistical analyses

Subject characteristics were reported as number (proportion) for categorical variables and means (standard deviation) for continuous variables. The differences between first and second cohort subjects were assessed using two sample t-tests for continuous variables and Chi-square or Fisher’s exact tests for categorical variables as appropriate. The characteristics of cases and controls in the first and second cohorts were also compared.

### Metabolome-wide association studies

Metabolome-wide association study (MWAS) was carried out for first and second cohort subjects separately by fitting individual Cox proportional hazards regression models for each feature with time to all-cause mortality (censored at 3 years) as the dependent variable and the feature intensity value as the independent variable. Cox models were adjusted for age, sex (male vs female), and race (black vs other). Batch effect was accounted for by treating batches as a categorical covariate in Cox models. Multiple hypothesis testing correction was performed using the Benjamini-Hochberg False Discovery Rate (FDR) method. [25]

### Metabolite annotation

Metabolite annotation was performed using a combination of computational methods, LC-MS/MS, and comparison of retention time with authentic standards. Computational metabolite annotation was performed using the R package xMSannotator (available at https://sourceforge.net/projects/xmsannotator/). xMSannotator uses a multilevel clustering procedure based on correlation of features, retention time, mass defect, isotope/adduct patterns, and network and pathway associations for categorizing database matches into different confidence levels. [26] Confidence levels range from zero to three, designating annotations from no to high confidence, which reduces the risk of false annotations and allows prioritization of computationally derived annotations for further experimental evaluation and confirmation using MS/MS and authentic standards. [26] Annotations with confidence score 2 or above from xMSannotator were targeted for MS/MS evaluation and the retention times were compared with an in-house library of previously confirmed metabolites. Metabolite identification levels were assigned using an identification scheme adapted from Schymanski et al. [27] A list of our in-house database of metabolites confirmed using MS/MS and authentic standards has been previously published. [11]

### Metabolic pathway analyses

In the first cohort, features associated with all-cause mortality that had an FDR-adjusted q-value<0.20 in MWAS were chosen as target features that entered pathway analysis in Mummichog (version 1.0.7). [28] The same approach was used in the second cohort in a separate analysis. For both first and second cohort pathway analyses, all features from first (n = 6,781) and second cohorts (n = 8,714) were utilized as the feature reference pool. The Mummichog output of significant pathways (p-value<0.05) identified in first and second cohorts was compared.

### Metabolomic score derivation

The metabolomic risk score was created by identifying common features in both cohorts that were associated with all-cause mortality and passed the FDR-adjusted threshold of q-value <0.20 in MWAS. We allowed for an m/z difference of 10 ppm and a retention time difference of 40 seconds when identifying common features. These common metabolites were selected as candidates for creating a metabolomic risk score and Least Absolute Shrinkage and Selection Operator (LASSO) regression was utilized to further sub-select metabolites and avoid redundency. [29] We evaluated the association of these sub-selected metabolites with circulating hs-CRP, hs-cTnI, and NT-proBNP levels using linear regression models adjusted for age, sex, race, and batch effect.

The hazard ratio (HR) estimate for the association of each feature selected after LASSO regression with all-cause mortality in the first cohort was natural log-transformed and the ln(HR) (i.e., coefficient estimate) of each feature was treated as its weight. The weighted metabolomic risk score was calculated as the sum of each feature’s intensity multiplied by its respective weight. The weighted score was mean centered, scaled by standard deviation, and categorized by its median and tertile cutoffs.

### Metabolomic score performance

The metabolomic risk score’s performance for predicting the primary outcome was first tested in the second cohort. Kaplan-Meir curves were utilized to evaluate the survival probability of subjects belonging to risk score categories by at the median and tertile cutoffs. Cox proportional hazards regression models adjusted for age, sex, race, and batch effect were utilized to test the score’s predictive value in the second cohort. As a sensitivity analysis, age was dichotomized at 75 years and Cox models were adjusted for current smoking, eGFR below 60 ml/min/1.73 m^2^, diagnosis of hypertension, diabetes, HF, PAD, stroke, and prior CABG. These variables were chosen because a recent study from the Thrombolysis In Myocardial Infarction (TIMI) group showed that a simple integer-based scheme using these predictors can stratify atherothrombotic risk in a secondary prevention population. [2] As an exploratory analysis, we further adjusted Cox models for log-transformed circulating levels of hs-CRP, hs-cTnI, and NT-proBNP.

The score’s all-cause mortality risk calibration capability in the second cohort was studied using a calibration plot and Hosmer-Lemeshow chi-square test. The risk discrimination and reclassification capabilities were studied in context of two baseline models: a) a model comprising of age, sex, and race; and b) a model comprising of age (dichotomized at 75 years), current smoking, eGFR below 60 ml/min/1.73 m^2^, hypertension, diabetes, HF, PAD, stroke, and prior CABG. Change c-statistic, Integrated Discrimination Index (IDI), and continuous Net Reclassification Index (NRI) after adding the metabolomic score to the two baseline models was evaluated. The features were further normalized using the ‘limma’ package prior to conducting these analyses. [30] The metabolomic score’s performance for predicting the secondary outcome of cardiovascular death was tested using the same approach outline above. Finally, the score’s performance was evaluated in the combined cohort as well. The workflow for this study is displayed in S2 Fig and the STROBE statement for this study is provided in S1 Table. All analyses were performed using R version 3.5.1 (R Foundation for Statistical Computing, Vienna, Austria). All relevant data are within the manuscript and its Supporting Information files.

## Results

### Baseline characteristics

The mean age of 454 subjects in the first cohort was 67.5 years, 65% were male, and 16% were black (Table 1). The second cohort included 322 subjects, with mean age of 65.9 years, 63% were male, and 19% were black (Table 1). Subjects in both cohorts had similar demographic characteristics, cardiovascular risk factor burden, prevalence of PAD, stroke, HF, and statin use. However, subjects in the first cohort had a higher prevalence of prior CABG and acute myocardial infarction (MI) at the time of enrolment (Table 1). The baseline characteristics of cases and controls in the first and second cohorts are described in S2 Table.

**Table 1:** Baseline characteristics of participants in the first and second cohorts.

Participant Characteristics	First cohort (n = 454)	Second cohort (n = 322)	p-value
Age, years	67.5 (10.9)	65.9 (11.6)	0.058
Men (%)	295 (65.0)	202 (62.7)	0.571
Black race (%)	71 (15.6)	60 (18.6)	0.317
Diabetes (%)	178 (39.2)	119 (37.2)	0.621
Hypertension (%)	306 (67.7)	230 (72.1)	0.219
Current smoking (%)	51 (11.2)	27 (8.4)	0.194
Body mass index, kg/m^2^	28.4 (5.7)	28.9 (6.7)	0.313
Estimated GFR, ml/min/1.73 m^2^	68.2 (23.4)	71.0 (23.9)	0.108
History of CABG (%)	162 (35.7)	88 (27.3)	0.014
History of PAD (%)	107 (23.6)	66 (20.5)	0.311
History of stroke (%)	49 (10.8)	40 (12.4)	0.483
History of heart failure (%)	158 (34.8)	118 (36.6)	0.642
Ejection fraction, %	51.5 (13.0)	52.7 (12.2)	0.217
Acute MI at presentation (%)	51 (11.2)	19 (5.9)	0.015
ACEi/ARB use (%)	316 (69.6)	168 (52.2)	<0.001
Aspirin use (%)	374 (82.4)	218 (67.7)	<0.001
Beta blocker use (%)	332 (73.1)	207 (64.3)	0.009
Clopidogrel use (%)	262 (57.7)	131 (40.7)	<0.001
Statin use (%)	346 (76.2)	212 (65.8)	0.002

Continuous variables described as mean (standard deviation) and categorical variables as count (proportion). Abbreviations: GFR = glomerular filtration rate, CABG = coronary artery bypass grafting, PAD = peripheral artery disease, MI = myocardial infarction, ACEi = angiotensin converting enzyme inhibitor, ARB = angiotensin-II receptor blocker.

### Metabolome-wide association studies

**Fig 1:**
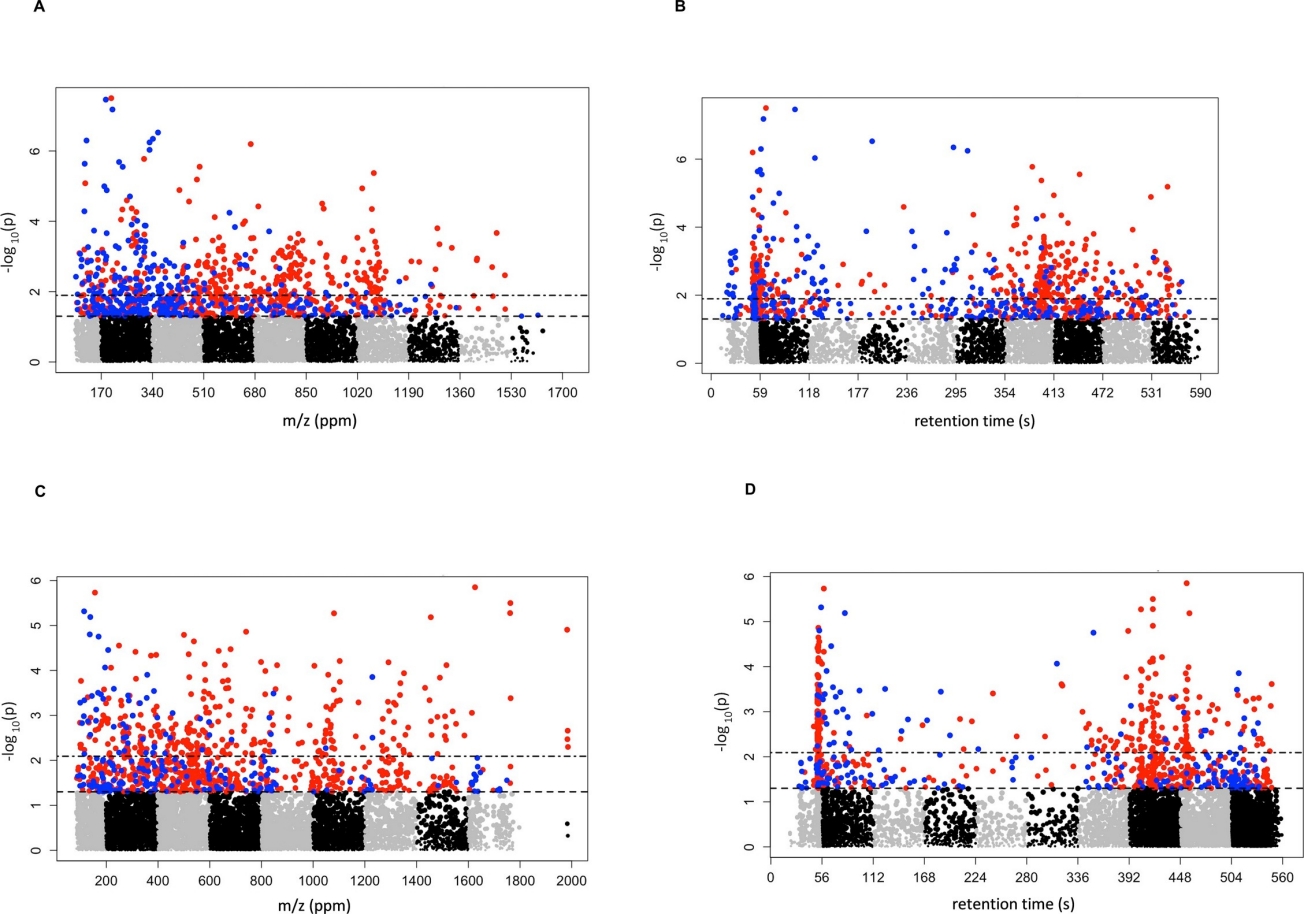
Manhattan plots for metabolome-wide association studies in the first and second cohorts. Each dot represents a unique feature and red dots are features with hazard ratio <1, while blue dots are features with hazard ratio >1. **Part A:** mass-to-charge (m/z) of features in first cohort plotted against -log_10_(p-value), **Part B:** retention time (s) of features in first cohort plotted against -log_10_(p), **Part C:** mass-to-charge (m/z) of features in second cohort plotted against -log_10_(p-value), and **Part D:** retention time (s) of features in second cohort plotted against -log_10_(p). Upper horizontal line depicts false discovery rate-adjusted q-value<0.2 and lower horizontal line depicts raw p-value<0.05.

A total of 6,781 metabolites were analyzed in the first cohort MWAS and at a raw p-value threshold of <0.05, 948 metabolites were independently associated with all-cause mortality (591 with HR<1 and 357 with HR>1) after adjusting for age, sex, and race. After FDR correction, 433 metabolites remained significantly associated with all-cause mortality (298 with HR<1 and 135 with HR>1) at the q-value threshold of <0.20. The Manhattan plots for this analysis are depicted in Fig 1A and 1B. The MWAS analysis was performed separately in the second cohort using 8,714 metabolites. At a raw p-value threshold of <0.05, 934 metabolites independently associated with the primary outcome (719 with HR<1 and 215 with HR>1) after adjusting for age, sex, and race. After FDR correction, 357 metabolites (299 with HR<1 and 57 with HR>1) passed the q-value threshold of <0.20 (Fig 1C and 1D).

### Metabolic pathway analyses

**Fig 2:**
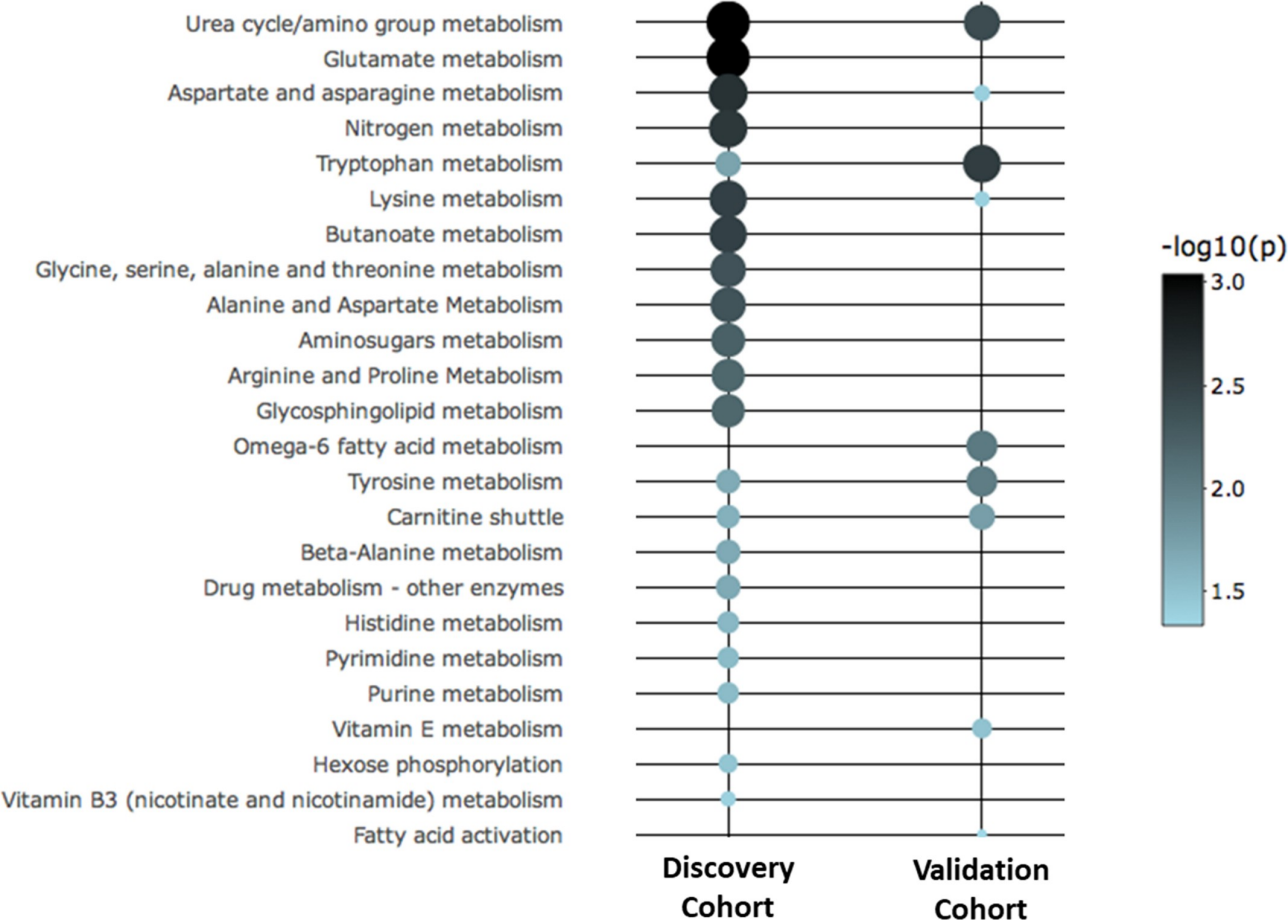
Bubble plot for metabolic pathways associated with all-cause mortality in the first and second cohorts. The color intensity of each bubble is inversely proportional to the p-value for the association of a metabolic pathway with all-cause mortality. Twenty-one pathways in the first cohort and nine pathways in the second cohort were associated with all-cause mortality. Six metabolic pathways were common between the two cohorts—urea cycle/amino group metabolism, tryptophan metabolism, aspartate/asparagine metabolism, lysine metabolism, tyrosine metabolism, and carnitine shuttle pathway.

Metabolic pathway analysis was conducted in the first cohort by using the 433 metabolites of significance as input features in Mummichog. Twenty-one metabolic pathways associated with all-cause mortality and the urea cycle/amino group metabolism pathway was the most significant (Fig 2 and S3 Table). In the second cohort, 357 metabolites of significance were used as input features and 9 metabolic pathways associated with all-cause mortality, among which the tryptophan metabolism pathway was the most significant (Fig 2 and S3 Table). On comparing significant metabolic pathways between the first and second cohorts there were six common metabolic pathways that were associated with all-cause mortality—urea cycle/amino group metabolism, tryptophan metabolism, aspartate/asparagine metabolism, lysine metabolism, tyrosine metabolism, and carnitine shuttle pathway (Fig 2).

### Metabolomic risk score derivation

Among the 433 and 357 metabolites that passed the FDR-adjusted q-value threshold of 0.20 during MWAS in the first and second cohorts, respectively, we observed that 24 metabolites associated with all-cause mortality were common between the two cohorts. These metabolites were selected for LASSO regression and a total of 7 metabolites which were not correlated with each other were selected as components of the metabolomic risk score (Table 2). Among these 7 metabolites two were annotated as creatinine [M+H] (13C) (m/z 115.0693, rt 55 s) and N8-Acetylspermidine (m/z 188.1755, rt 51 s). Three metabolites were associated with an increased hazard of mortality and four metabolites were associated with a decreased hazard of mortality in both the cohorts (Table 2).

**Table 2:** Unique metabolic features comprising the metabolomic risk score.

		First Cohort			Second Cohort		
*m/z (ppm)*	*rt* (s)	HR	95% CI	q-value	HR	95% CI	q-value
115.0693*	55	1.62	(1.33,1.98)	0.001	1.58	(1.22, 2.03)	0.052
188.1755^¶^	51	2.27	(1.57, 3.29)	0.004	3.01	(1.63, 5.56)	0.051
207.1106	65	1.74	(1.42, 2.13)	<0.001	1.77	(1.35, 2.32)	0.018
444.6726	55	0.74	(0.61, 0.90)	0.076	0.81	(0.69, 0.95)	0.198
559.2977	388	0.65	(0.50, 0.83)	0.047	0.66	(0.49, 0.89)	0.170
1050.6578	412	0.76	(0.63, 0.92)	0.107	0.73	(0.61, 0.87)	0.060
1078.627	412	0.74	(0.63, 0.88)	0.038	0.72	(0.60, 0.87)	0.058

Abbreviations: HR = hazard ratio, CI = confidence interval, m/z = mass-to-charge, rt = retention time; s = seconds. Metabolite identified as *Creatinine[M+H] (13C) and ^¶^N8-Acetylspermidine

The association of these 7 metabolites with cardiovascular biomarkers was explored using linear regression models (S4 Table), The metabolites associated with an increased mortality hazard were also associated with higher circulating levels of hs-cTnI and NT-proBNP, and vice versa (S4 Table). However, the associations with hs-CRP were inconsistent.

### Metabolomic risk score performance

**Fig 3:**
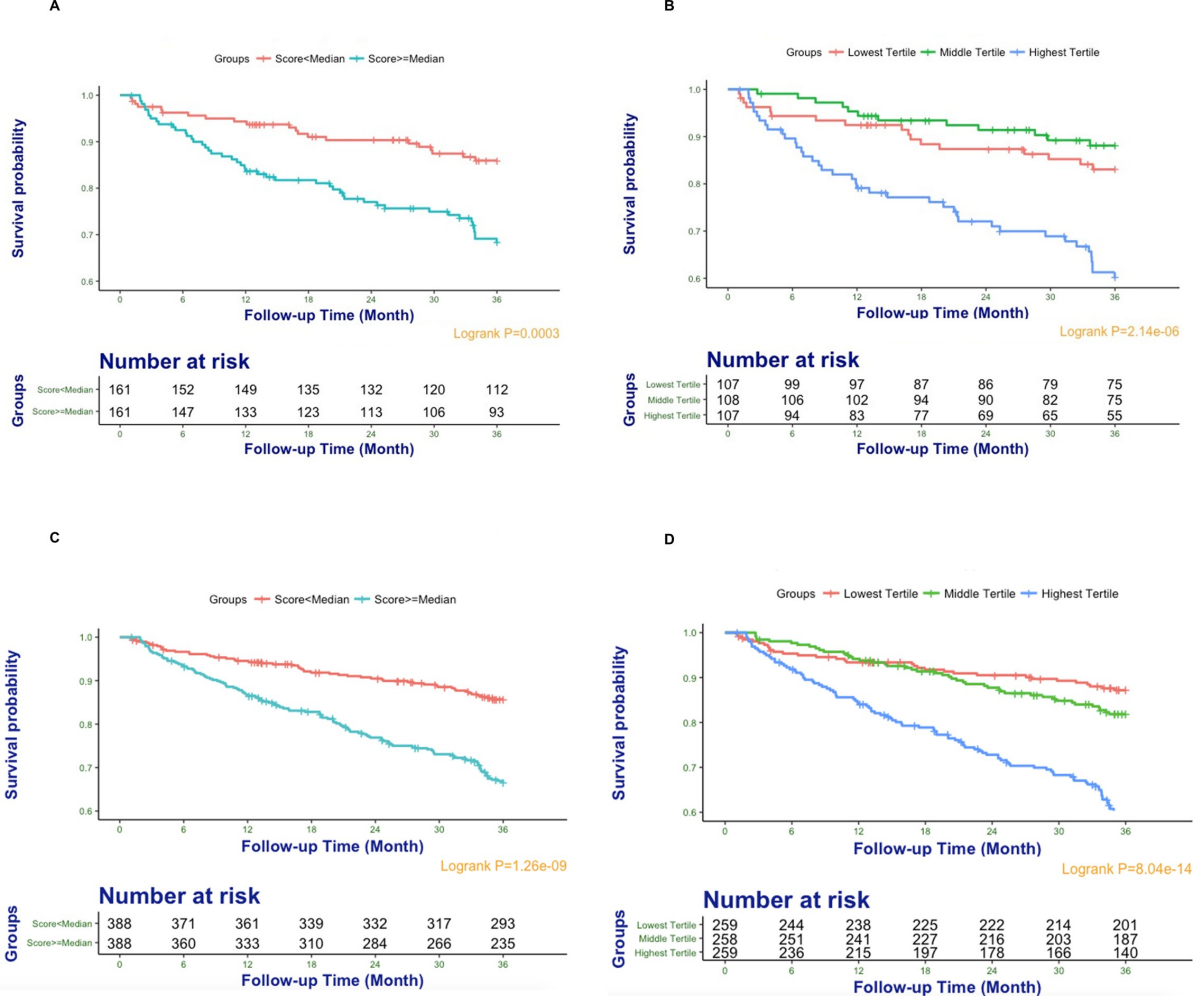
Kaplan–Meier survival curves for subjects stratified using metabolomic risk score categories. Subjects with metabolomic risk score value above the median were at a higher risk of all-cause mortality as compared with those with a score below the median in both the second cohort (**Part A**) and the combined cohort (**Part C**). Subjects in second (**Part B**) and combined cohort (**Part D**) with metabolomic risk score value in the highest tertile were at a higher risk of all-cause mortality as compared with those with a score in the first two tertiles.

The metabolomic risk score’s values ranged from -6.203 to 4.933 with median, lower tertile, and upper tertile cut-off values of -0.075, -0.394, and 0.255, respectively. Subjects in the second cohort with a score greater than the median experienced an increased risk of all-cause mortality as compared to those with a score below the median (Fig 3A). This difference was primarily driven by subjects in the highest tertile group that were at a significantly higher risk of the primary outcome as compared with their counterparts in the lower two tertiles (Fig 3B). Similar findings were observed in the combined cohort (Fig 3C and 3D) as well as for the secondary outcome of cardiovascular death (S3A–S3D Fig).

The metabolomic risk score was associated with all-cause mortality in the second cohort with a HR of 2.26 (95% CI 1.7, 2.90; p<0.001) per 1-unit increase, after adjusting for age, sex, race, and batch effect (Table 3). Subjects with a metabolomic score value above the median were at a three-fold risk of all-cause mortality as compared to those with score value below the median (Table 3). Age above 75 years was the only risk factor independently associated with all-cause mortality (HR 2.12, 95% CI 1.21, 3.71; p = 0.009) in the model containing variables evaluated in the recent TIMI study. Nevertheless, the risk score retained its independent predictive value after multivariable adjustment (Table 3). Similarly, the risk score was associated with the primary outcome in the combined cohort after adjusting for age, sex, race, and batch effect (Table 3). Current smoking was the only risk factor independently associated with all-cause mortality (HR 2.29, 95% CI 1.38, 3.80; p = 0.001) in the multivariable adjusted model. The risk score retained its independent predictive value after multivariable adjustment in the combined cohort as well (Table 3). Furthermore, adjustment for hs-CRP, hs-cTnI, and NT-proBNP levels did not attenuate this association (S5 Table). The metabolomic risk score was also associated with the secondary outcome of cardiovascular death in both the second and combined cohorts (S6 Table).

**Table 3:** Association of metabolomic risk score with all-cause mortality in the second and combined cohorts.

	Second cohort		Combined cohort	
	HR (95% CI)	p-value	HR (95% CI)	p-value
Per 1-SD increase				
Unadjusted	2.16 (1.69, 2.76)	<0.001	2.18 (1.85, 2.56)	<0.001
Model 1*	2.26 (1.76, 2.90)	<0.001	2.19 (1.86, 2.59)	<0.001
Model 2^¶^	2.14 (1.62, 2.83)	<0.001	2.00 (1.66, 2.42)	<0.001
Above/Below Median^†^				
Unadjusted	3.00 (1.68, 5.36)	<0.001	3.02 (2.14, 4.28)	<0.001
Model 1*	3.00 (1.65, 5.49)	<0.001	2.91 (2.05, 4.12)	<0.001
Model 2^¶^	2.39 (1.27, 4.50)	<0.001	2.38 (1.63, 3.46)	<0.001

* Model 1 adjusted for age, sex, race, and batch effect.

^¶^ Model 2 adjusted for age (dichotomized at 75 years), current smoking, hypertension, diabetes, HF, PAD, stroke, prior CABG, eGFR (dichotomized at 60 ml/min/1.73 m^2^), and batch effect.

^†^Individuals with lower than median metabolomic risk score are the reference group. Abbreviations: HR = hazard ratio, CI = confidence interval, and SD = standard deviation.

The risk score was well-calibrated in the second cohort with Hosmer-Lemeshow chi-square p = 0.84 (S4 Fig). Furthermore, adding the risk score to the baseline model comprising of age, sex, and race as well as the model containing TIMI risk factors resulted in a nominal improvement in C-statistic in the second cohort (Table 4). However, in the combined cohort addition of the risk score to both models resulted in a significant improvement in the C-statistic (Table 4). Additionally, the risk score resulted in a significant improvement in IDI and a significant change in continuous NRI for both models in the second and combined cohorts (Table 4). Similar findings were observed for the secondary outcome as well (S7 Table).

**Table 4:** Improvement in all-cause mortality risk discrimination and risk reclassification indices with metabolomic risk score.

	Second cohort		Combined cohort	
	Estimate (95% CI)	p-value	Estimate (95% CI)	p-value
Model 1*				
Baseline C-statistic	0.618 (0.527, 0.710)	-	0.574 (0.515, 0.634)	-
Delta C-statistic	0.066 (-0.002, 0.135)	-	0.104 (0.045, 0.163)	-
IDI	0.109 (0.049, 0.177)	<0.001	0.097 (0.059, 0.143)	<0.001
NRI	21.2% (5.8%, 35.3%)	0.006	26.3% (18.2%, 35.0%)	<0.001
Model 2^¶^				
Baseline C-statistic	0.678 (0.608, 0.748)	-	0.653 (0.604, 0.703)	-
Delta C-statistic	0.039 (-0.006, 0.086)	-	0.047 (0.016, 0.078)	-
IDI	0.084 (0.030, 0.151)	0.002	0.058 (0.026, 0.095)	<0.001
NRI	23.3% (7.9%, 38.2%)	0.006	19.9% (11.2%, 29.4%)	<0.001

* Model 1 consists of age, sex, and race.

^¶^ Model 2 consists of age (dichotomized at 75 years), current smoking, hypertension, diabetes, HF, PAD, stroke, prior CABG, and eGFR (dichotomized at 60 ml/min/1.73 m^2^). Abbreviations: CI = confidence interval, IDI = Integrated Discrimination Index, and NRI = Net Reclassification Index.

## Discussion

We report two important findings in this study of subjects with CAD undergoing untargeted high-resolution plasma metabolomic profiling. First, differential regulation of six validated metabolic pathways was associated with all-cause mortality in our cohort. Second, a novel metabolomic risk score was created using seven unique metabolites that was highly predictive of adverse cardiovascular outcomes, improved risk reclassification, and added incremental risk discriminatory value to a risk factor model comprising of traditional cardiovascular risk factors.

Our results indicate that six metabolic pathways (urea cycle/amino group metabolism, tryptophan metabolism, aspartate/asparagine metabolism, lysine metabolism, tyrosine metabolism, and the carnitine shuttle) involved in myocardial energetics and inflammation are associated with mortality among patients with CAD. CAD is a complex condition characterized by inflammation, arterial remodeling and metabolic reprogramming at the level of cardiac myocytes. [31, 32] In this regard, clinical second of the associated metabolic phenotype provides an opportunity to elucidate the end-products of genetic and environmental factors that drive clinical outcomes among patients with CAD. [33] Herein, we have used computational pathway analyses to investigate mechanistic biological pathways that are associated with increased mortality in our cohort. By shifting the focus to metabolite clusters with a collective biological function, our analytical technique reduces false positive findings, improves reproducibility between independent studies, and elucidates potential culprit pathways for preclinical focus. [28, 34]

Under normal conditions, the myocardium derives 50–70% of its energy requirement from mitochondrial β-oxidation of fatty acids. [35–37] However, during ischemia, energy demand-supply mismatch and intracellular acidification results in a metabolic shift to anaerobic, non-glycolytic amino acid substrates that are able to replenish Kreb’s cycle intermediates through a process known as anaplerosis. [38–42] We found that key metabolic pathways—the carnitine shuttle, lysine, aspartate and asparagine, and urea cycle metabolism, representative of this ischemic metabolic adaptation are associated with all-cause mortality in our cohort. Acylcarnitines are lipid intermediates that shuttle fatty acids into mitochondria for β-oxidation, [43] and several prior studies have demonstrated that impaired carnitine shuttle function and elevated acylcarnitine levels predict adverse cardiovascular outcomes and death in CAD patients. [44–47] We were able to validate these findings in our cohort in addition to other novel metabolites.

Our results also indicate that differential regulation of lysine, aspartate and asparagine, and urea cycle is associated with mortality among patients with CAD. 6-N-trimethyllysine (TML) is a precursor in carnitine biosynthesis, [48, 49] however few studies have focused on the role of lysine metabolism in prognosticating CAD outcomes. Loland and colleagues showed that baseline TML levels are associated with angiographic CAD progression in a study of 183 patients. [50] Aspartate and asparagine are key metabolic sinks for excess Kreb’s cycle intermediates produced during anaplerosis. [39] Preclinical studies have shown that inhibition of aspartate utilization reduces ischemic resilience, [51] and that inclusion of aspartate in cardioplegic solutions improves post-ischemic recovery. [52, 53] Nevertheless, these findings have not yet been translated into prognostication of adverse outcomes in CAD patients. The urea cycle functions to excrete excess ammonia generated from amino acid catabolism. [54] Prior studies by Shah et al. [46] and Amin et al. [55] demonstrated that patients with CAD differentially regulate urea cycle metabolism compared to healthy controls.

Dysregulated tyrosine and tryptophan metabolic pathways were also predictive of mortality in our cohort. Interferon gamma (IFN-γ), an inflammatory cytokine that plays a key role in the development and progression of CAD [56–59] regulates tryptophan and tyrosine metabolism. Tryptophan is catabolized to kynurenine by Indoleamine 2,3-Dioxygenase, a rate limiting enzyme that is induced by IFN-γ. [60–66] The resultant high kynurenine:tryptophan ratio has previously been shown to correlate with the presence of CAD, [67] and to predict major coronary events and all-cause mortality in stable CAD patients. [61, 68] Tyrosine, a precursor for catecholamine biosynthesis, is synthesized from phenylalanine via phenylalanine hydroxylase (PAH). [69] Oxidative stress in IFN-γ stimulated macrophages reduces PAH bioavailability, diminishing tyrosine production. [70–72] In a large cohort of CAD patients, Murr et al. [72] showed that elevated Phenylalanine:Tyrosine ratios correlate with high sensitivity CRP levels, a well-established biomarker of adverse cardiovascular outcomes. [73, 74] Alterations in catecholamine biosynthesis and consequent neurohormonal dysregulation could represent an additional mechanism by which dysregulated tyrosine metabolism affects mortality in CAD patients. [75–77]

From a risk prediction perspective, it is well established that patients with stable CAD vary in their risk of mortality and personalizing risk assessment by identifying those at high-risk of adverse outcomes has been an area of active research interest. The TIMI risk score for secondary prevention predicts adverse outcomes among patients with known atherosclerotic vascular disease. [2] This risk score consists of nine easily measurable clinical variables (age, current smoking, hypertension, diabetes, heart failure, stroke, prior coronary artery bypass grafting, peripheral artery disease, and chronic kidney disease) and has been shown to perform well in external validation studies. [78, 79] However, this approach does not identify individual plasma metabolites or the dysregulated metabolic pathways associated with mortality in this high-risk patient population. In addition to identifying the six significant metabolic pathways described above, we identified a unique subset of seven metabolites was identified using stringent statistical criteria and these were used to create a novel metabolomic risk score. This risk score independently predicted mortality with each absolute unit increase in its value. This independent association was not attenuated after adjusting for cardiovascular biomarkers that represent systemic inflammation (hs-CRP), [80] subclinical myocardial injury (hs-cTnI), [81] or myocyte stretch (NT-proBNP), [82] and can be useful for prognosticating outcomes among patients with CAD. [83] Furthermore, adding the risk score to a model containing TIMI risk factors improved risk discrimination and reclassification indices. Once externally validated, this novel risk score may serve as an important tool for personalizing risk assessment among patients with CAD.

### Strengths and limitations

Strengths of our study include a high-risk patient population analyzed as two independent cohorts. Our untargeted high-resolution plasma metabolomic profiling technique is unique and the measurement of each metabolite in triplicate make our observations robust. Finally, the significant metabolic pathways and individual metabolites identified in our study along with the novel metabolomic risk score were internally validated.

Limitations of the study include a modest sample size and the lack of annotation for several unique metabolites associated with mortality. However, we have ascertained validated metabolic pathways that contain these metabolites and thus used them in our analysis. Our cohort consists of subjects living in Southeastern US and thus our findings may not be generalizable to other geographical regions. Most importantly, our findings pertaining to metabolic pathways and the risk predictive value of metabolomic risk need replication in external cohorts.

## Conclusions

The differential regulation of six metabolic pathways involved in myocardial energetics and inflammation, identified using untargeted high-resolution plasma metabolomic profiling is associated with all-cause mortality among patients with CAD. A novel metabolomic risk score consisting of metabolites associated with all-cause mortality in our cohort is highly predictive of mortality and holds promise for serving as a tool that prognosticates outcomes in this high-risk patient population.

## Supporting information

S1 Fig Principal component analysis plots of study samples and quality control samples. (DOCX)

S2 Fig Study design. (DOCX)

S3 Fig A. Kaplan–Meier survival curves for cardiovascular death among second cohort subjects stratified using metabolomic risk score categories above and below median. B. Kaplan–Meier survival curves for cardiovascular death among second cohort subjects stratified using metabolomic risk score categories of highest, middle, and lowest tertile. C. Kaplan–Meier survival curves for cardiovascular death among all subjects stratified using metabolomic risk score categories above and below median. D. Kaplan–Meier survival curves for cardiovascular death among all subjects stratified using metabolomic risk score categories of highest, middle, and lowest tertile.
(DOCX)

S4 Fig Metabolomic risk score calibration in the second cohort. (DOCX)

S1 Table STROBE statement. (DOCX)

S2 Table Baseline characteristics of cases and controls in the first and second cohorts. (DOCX)

S3 Table Metabolic pathways associated with all-cause mortality in the first and second cohorts. (DOCX)

S4 Table Association of significant metabolites and metabolomic risk score with cardiovascular biomarkers. (DOCX)

S5 Table Association of metabolomic risk score with death after adjustment for cardiovascular biomarkers in the second and combined cohorts. (DOCX)

S6 Table Association of metabolomic risk score with cardiovascular death in the second and combined cohorts. (DOCX)

S7 Table Improvement in cardiovascular death risk discrimination and risk reclassification indices with metabolomic risk score*. (DOCX)

## Abbreviations

AHA: American Heart Association
BMI: Body Mass Index
CAD: Coronary Artery Disease
CABG: Coronary Artery Bypass Graft
eGFR: estimated Glomerular Filtration Rate
FDR: False Discovery Rate
HF: Heart Failure
HR: Hazard Ratio
Hs-CRP: high-sensitivity C-reactive protein
Hs-cTnI: high-sensitivity cardiac Troponin-I
ICD: International Classification of Diseases
IDI: Integrated Discrimination Index
IFN-γ: Interferon-gamma
LASSO: Least Absolute Shrinkage and Selection Operator
LC/MS: Liquid Chromatography/Mass Spectrometry
LVEF: Left Ventricular Ejection Fraction
μL: microliter
MWAS: Metabolome Wide Association Study
m/z: mass/charge
NIH: National Institutes of Health
NHLBI: National Heart, Lung, and Blood Institute
NRI: Net Reclassification Index
NT-proBNP: N-terminal prohormone of Brain Natriuretic Peptide
PAD: Peripheral Artery Disease
PAH: phenylalanine hydroxylase
rt: retention time
SD: standard deviation
TIMI: Thrombolysis In Myocardial Infarction
TML: N-trimethyllysine
USA: United States of America
