# Supplementary figures and images for: Untargeted high-resolution plasma metabolomic profiling predicts outcomes in patients with coronary artery disease
Source: PLoS One. 2020 Aug 18;15(8):e0237579. doi: 10.1371/journal.pone.0237579 (PMC7444579; doi:10.1371/journal.pone.0237579)

**S1 Figure: Principal Component Analysis plots of study samples and quality control samples**


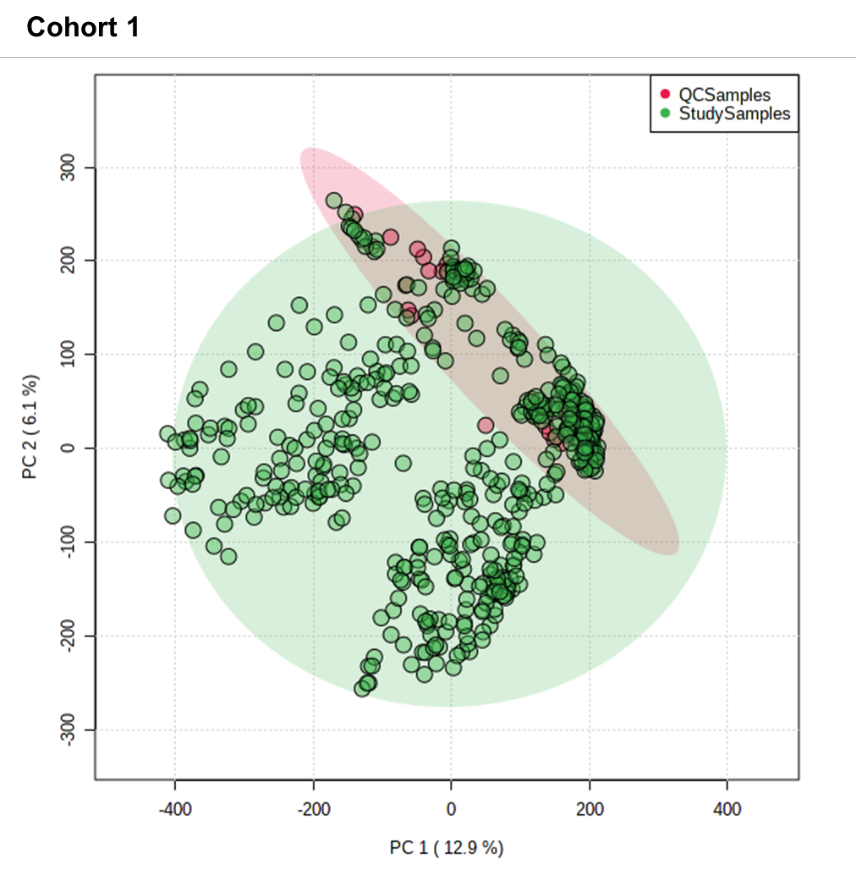


**
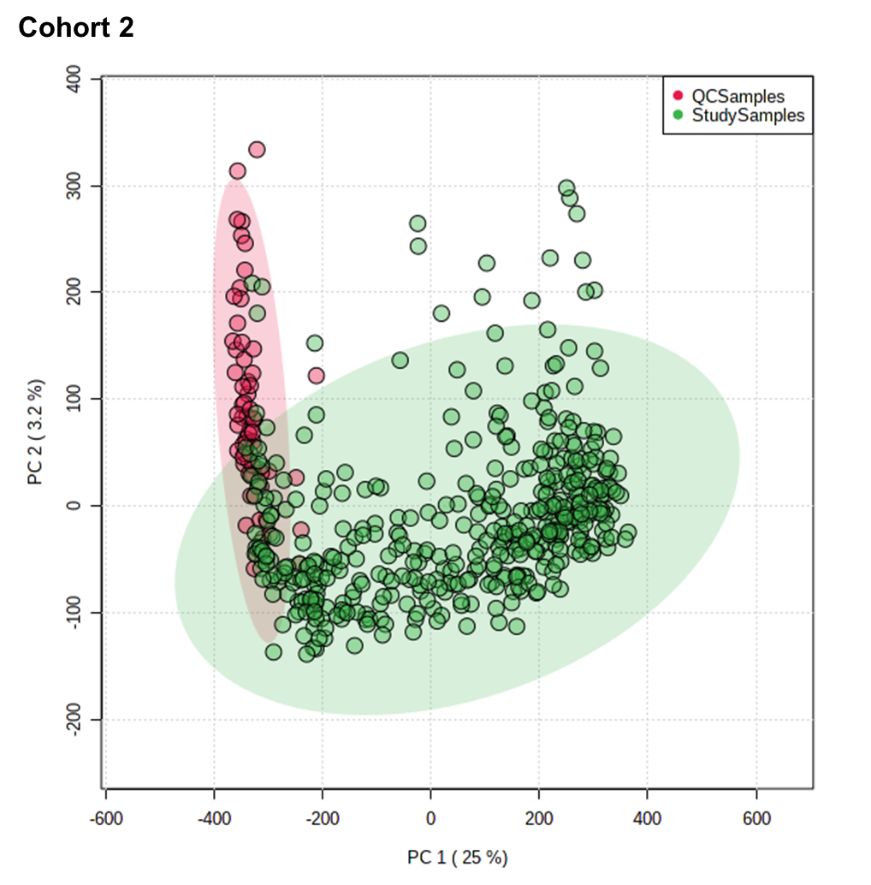
**

Supplement: S1 Fig — (DOCX) [file pone.0237579.s001.docx]

**
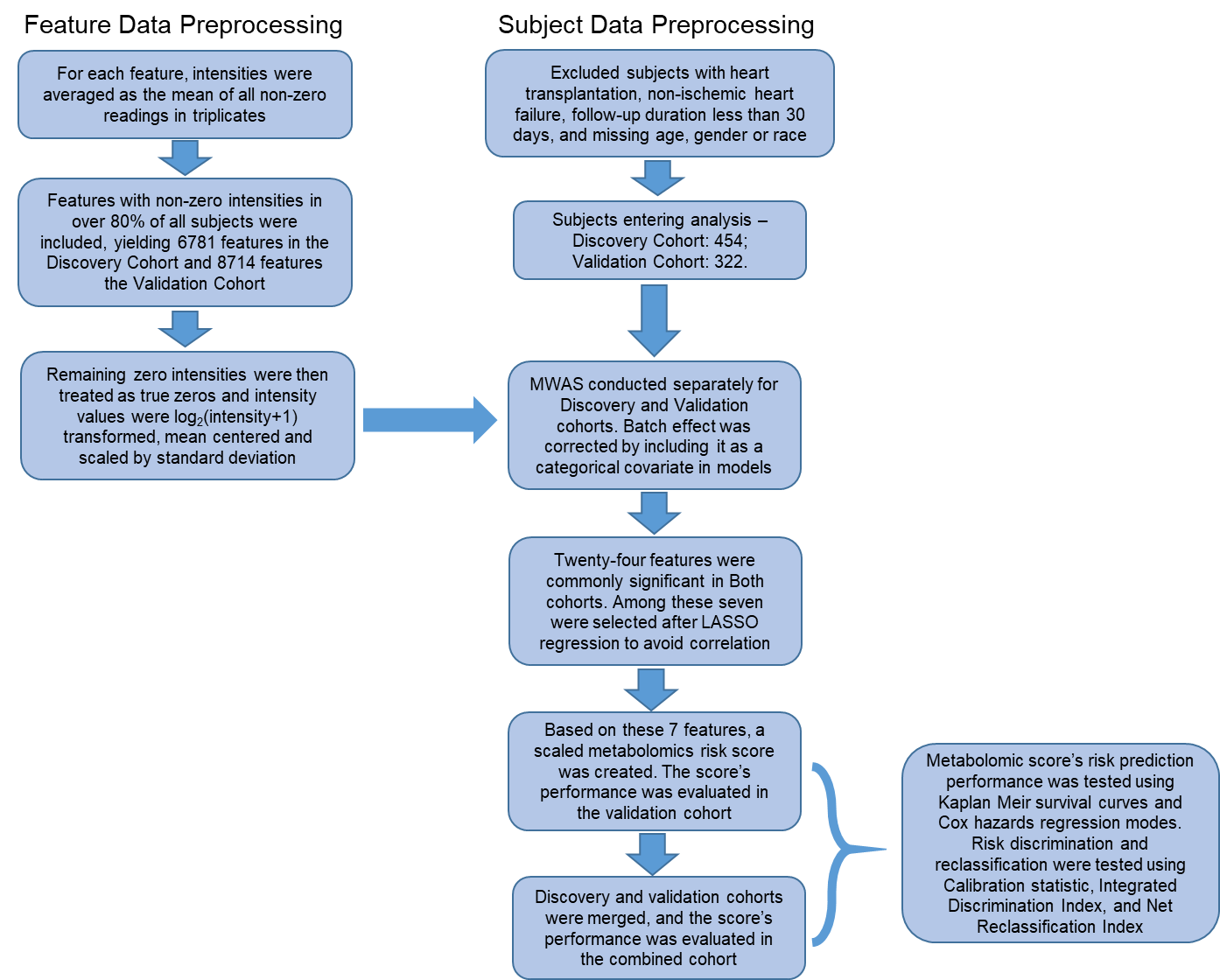
S2 Figure: Study design**

Supplement: S2 Fig — (DOCX) [file pone.0237579.s002.docx]

**S4 Figure: Metabolomic risk score calibration in the second cohort**


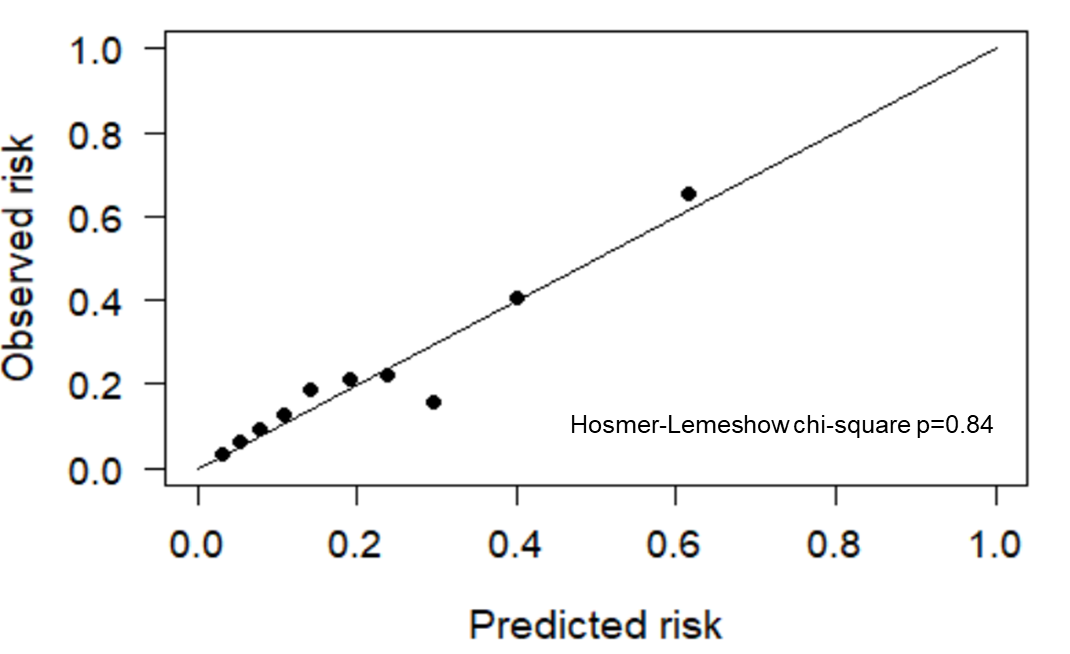

Supplement: S4 Fig — (DOCX) [file pone.0237579.s004.docx]
