# Supplementary material for: Untargeted high-resolution plasma metabolomic profiling predicts outcomes in patients with coronary artery disease
Source: PLoS One. 2020 Aug 18;15(8):e0237579. doi: 10.1371/journal.pone.0237579 (PMC7444579; doi:10.1371/journal.pone.0237579)
Supplement: S3 Fig — A. Kaplan–Meier survival curves for cardiovascular death among second cohort subjects stratified using metabolomic risk score categories above and below median. B. Kaplan–Meier survival curves for cardiovascular death among second cohort subjects stratified using metabolomic risk score categories of highest, middle, and lowest tertile. C. Kaplan–Meier survival curves for cardiovascular death among all subjects stratified using metabolomic risk score categories above and below median. D. Kaplan–Meier survival curves for cardiovascular death among all subjects stratified using metabolomic risk score categories of highest, middle, and lowest tertile. (DOCX) [file pone.0237579.s003.docx]

**S3A Figure: Kaplan–Meier survival curves for cardiovascular death among second cohort subjects stratified using metabolomic risk score categories above and below median**


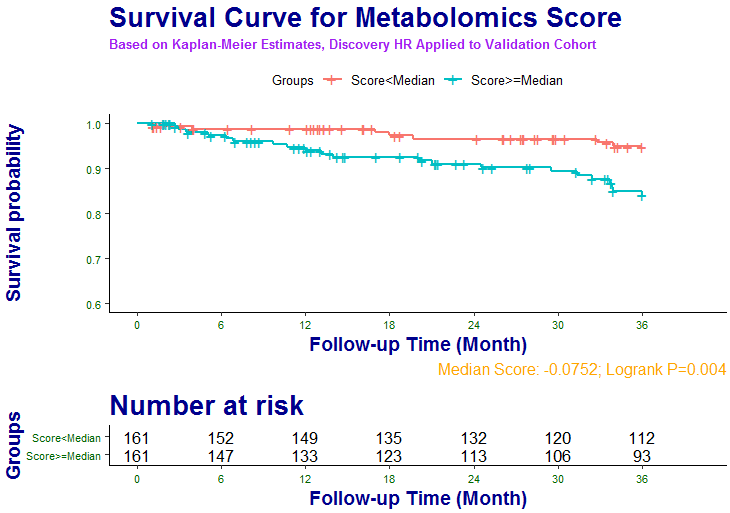


**S3B Figure: Kaplan–Meier survival curves for cardiovascular death among second cohort subjects stratified using metabolomic risk score categories of highest, middle, and lowest tertile**


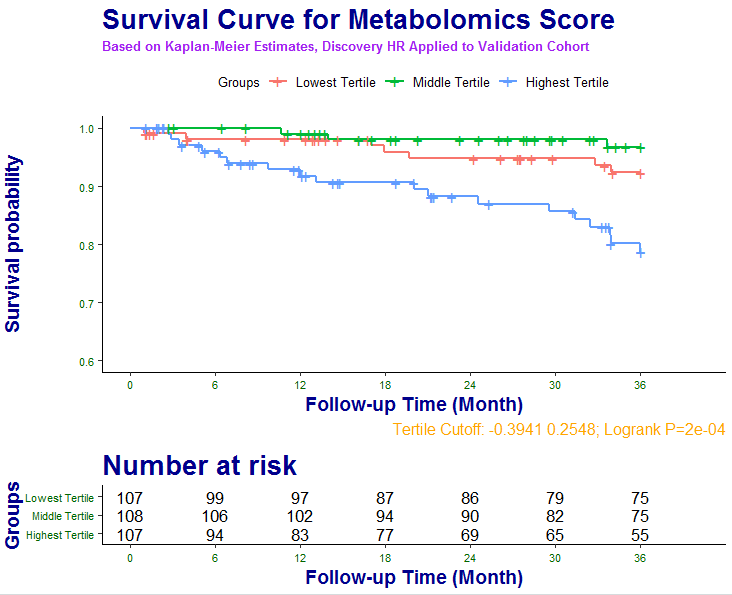


**S3C Figure: Kaplan–Meier survival curves for cardiovascular death among all subjects stratified using metabolomic risk score categories above and below median**


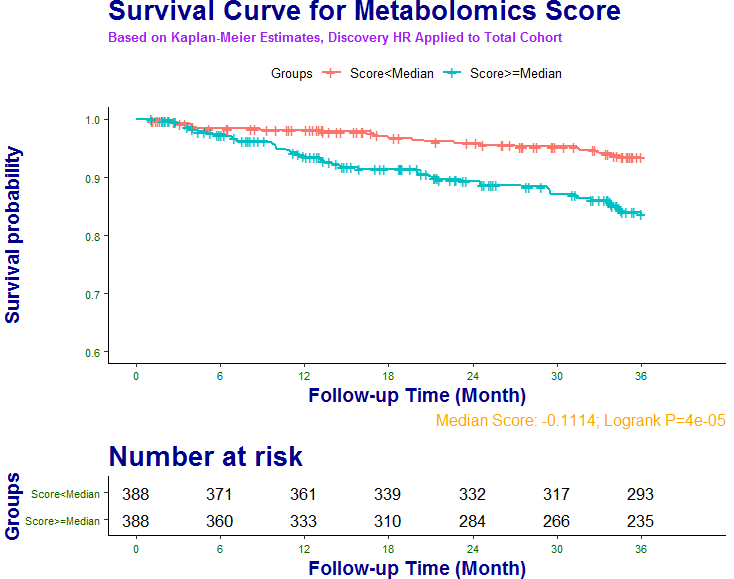


**S3D Figure: Kaplan–Meier survival curves for cardiovascular death among all subjects stratified using metabolomic risk score categories of highest, middle, and lowest tertile**


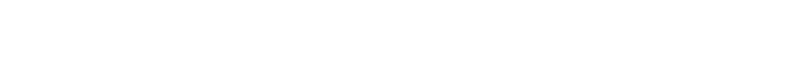

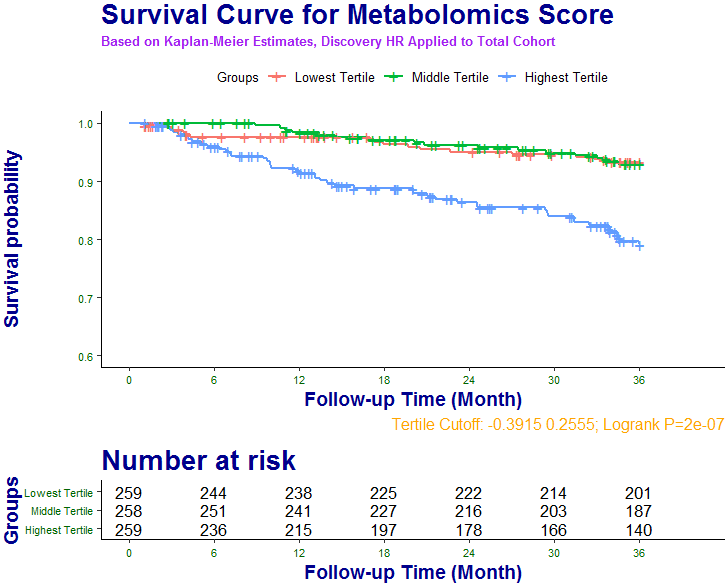


1. Schymanski EL, Jeon J, Gulde R, Fenner K, Ruff M, Singer HP and Hollender J. Identifying small molecules via high resolution mass spectrometry: communicating confidence. *Environ Sci Technol*. 2014;48:2097-8.
