# Supplementary material for: Untargeted high-resolution plasma metabolomic profiling predicts outcomes in patients with coronary artery disease
Source: PLoS One. 2020 Aug 18;15(8):e0237579. doi: 10.1371/journal.pone.0237579 (PMC7444579; doi:10.1371/journal.pone.0237579)
Supplement: S1 Table — (DOCX) [file pone.0237579.s005.docx]

**S1 Table. STROBE Statement**

|  | Item No. | Recommendation | Page No. |
| --- | --- | --- | --- |
| Title and abstract | 1 | (a) Indicate the study’s design with a commonly used term in the title or the abstract | 2 |
|  |  | (b) Provide in the abstract an informative and balanced summary of what was done and what was found | 2 |
| Introduction |  |  |  |
| Background/  rationale | 2 | Explain the scientific background and rationale for the investigation being reported | 4 |
| Objectives | 3 | State specific objectives, including any prespecified hypotheses | 4-5 |
| Methods |  |  |  |
| Study design | 4 | Present key elements of study design early in the paper | 5-8 |
| Setting | 5 | Describe the setting, locations, and relevant dates, including periods of recruitment, exposure, follow-up, and data collection | 5,7,8 |
| Participants | 6 | (a) Give the eligibility criteria, and the sources and methods of selection of  participants. Describe methods of follow-up | 5,6,8 |
|  |  | (b) For matched studies, give matching criteria and number of exposed and unexposed | 5 |
| Variables | 7 | Clearly define all outcomes, exposures, predictors, potential confounders, and effect modifiers. Give diagnostic criteria, if applicable | 7,8,10 |
| Data sources/  measurement | 8 | For each variable of interest, give sources of data and details of methods of assessment (measurement).* Describe comparability of assessment methods if there is more than one group (*Give information separately for exposed and unexposed) | 6,7 |
| Bias | 9 | Describe any efforts to address potential sources of bias | 10,11 |
| Study size | 10 | Explain how the study size was arrived at | 6 |
| Quantitative variables | 11 | Explain how quantitative variables were handled in the analyses. If applicable, describe which groupings were chosen and why | 9-11 |
| Statistical methods | 12 | (a) Describe all statistical methods, including those used to control for confounding | 5-11 |
|  |  | (b) Describe any methods used to examine subgroups and interactions | NA |
|  |  | (c) Explain how missing data were addressed | NA |
|  |  | (d) If applicable, explain how loss to follow-up was addressed | NA |
|  |  | (e) Describe any sensitivity analyses | 10 |
| Results |  |  |  |
| Participants | 13 | (a) Report numbers of individuals at each stage of study—e.g. numbers potentially eligible, examined for eligibility, confirmed eligible, included in the study, completing follow-up, and analyzed | 6 |
|  |  | (b) Give reasons for non-participation at each stage | 6 |
|  |  | (c) Consider use of a flow diagram | NA |
| Descriptive data | 14 | (a) Give characteristics of study participants (e.g. demographic, clinical, social) and information on exposures and potential confounders | 22 |
|  |  | (b) Indicate number of participants with missing data for each variable of interest | 6 |
|  |  | (c) Summarize follow-up time (e.g., average and total amount) | 7 |
| Outcome data | 15 | Report numbers of outcome events or summary measures over time | 11 |
| Main results | 16 | (a) Give unadjusted estimates and, if applicable, confounder-adjusted estimates and their precision (e.g., 95% confidence interval). Make clear which confounders were adjusted for and why they were included | 23 |
|  |  | (b) Report category boundaries when continuous variables were categorized | 13 |
|  |  | (c) If relevant, consider translating estimates of relative risk into absolute risk for a meaningful time period | NA |
| Other analyses | 17 | Report other analyses done—e.g. analyses of subgroups and interactions, and sensitivity analyses | 14 |
| Discussion |  |  |  |
| Key results | 18 | Summarize key results with reference to study objectives | 14 |
| Limitations | 19 | Discuss limitations of the study, taking into account sources of potential bias or  imprecision. Discuss both direction and magnitude of any potential bias | 17 |
| Interpretation | 20 | Give a cautious overall interpretation of results considering objectives, limitations, multiplicity of analyses, results from similar studies, and other relevant evidence | 14-18 |
| Generalizability | 21 | Discuss the generalizability (external validity) of the study results | 17-18 |
| Other information |  |  |  |
| Funding | 22 | Give the source of funding and the role of the funders for the present study and, if applicable, for the original study on which the present article is based | 19 |
