# Supplementary material for: Untargeted high-resolution plasma metabolomic profiling predicts outcomes in patients with coronary artery disease
Source: PLoS One. 2020 Aug 18;15(8):e0237579. doi: 10.1371/journal.pone.0237579 (PMC7444579; doi:10.1371/journal.pone.0237579)
Supplement: S2 Table — (DOCX) [file pone.0237579.s006.docx]

**S2 Table: Baseline characteristics of cases and controls in the first and second cohorts**

|  | **First cohort (N=454)** | | | **Second cohort (N=322)** | | |
| --- | --- | --- | --- | --- | --- | --- |
| **Participant Characteristics** | **Controls**  **N=220** | **Cases**  **N=234** | **p-value** | **Controls**  **N=202** | **Cases**  **N=120** | **p-value** |
| Age, years | 65.1 (10.2) | 69.7 (11.0) | <0.001 | 65.2 (11.1) | 68.9 (11.8) | <0.001 |
| Men (%) | 144 (65.5) | 151 (64.5) | 0.845 | 121 (59.9) | 61 (67.5) | 0.191 |
| Black race (%) | 33 (15.0) | 38 (15.6) | 0.796 | 40 (19.8) | 20 (16.7) | 0.555 |
| Diabetes (%) | 73 (33.2) | 105 (44.9) | 0.012 | 72 (35.6) | 47 (39.8) | 0.474 |
| Hypertension (%) | 136 (62.4) | 170 (72.6) | 0.021 | 138 (68.7) | 92 (78.0) | 0.093 |
| Current smoking (%) | 27 (12.3) | 24 (10.3) | 0.553 | 17 (8.4) | 10 (8.3) | 1.000 |
| Body mass index, kg/m^2^ | 29.2 (4.8) | 28.3 (5.0) | 0.078 | 30.2 (7.0) | 26.9 (5.3) | <0.001 |
| Estimated GFR, ml/min/1.73 m^2^ | 76.2 (18.3) | 60.7 (25.2) | <0.001 | 77.4 (20.2) | 60.2 (25.6) | <0.001 |
| History of CABG (%) | 61 (27.7) | 101 (43.2) | 0.001 | 44 (21.8) | 44 (36.7) | 0.004 |
| History of PAD (%) | 37 (16.8) | 70 (29.9) | 0.001 | 31 (15.3) | 35 (29.2) | 0.004 |
| History of stroke (%) | 21 (9.5) | 28 (12.0) | 0.451 | 20 (9.9) | 20 (16.7) | 0.082 |
| History of heart failure (%) | 50 (22.7) | 108 (46.2) | <0.001 | 59 (29.2) | 59 (49.2) | <0.001 |
| Ejection fraction, % | 54.6 (9.2) | 50.0 (14.7) | <0.001 | 54.3 (10.1) | 49.1 (13.9) | 0.002 |
| Acute MI at presentation (%) | 24 (10.9) | 27 (11.5) | 0.882 | 9 (4.5) | 10 (8.3) | 0.220 |
| ACEi/ARB use (%) | 155 (70.5) | 161 (68.8) | 0.760 | 107 (53.0) | 61 (50.8) | 0.730 |
| Aspirin use (%) | 186 (84.5) | 788 (80.3) | 0.268 | 133 (65.8) | 85 (70.8) | 0.389 |
| Beta blocker use (%) | 163 (74.1) | 169 (72.2) | 0.673 | 131 (64.9) | 76 (63.3) | 0.811 |
| Clopidogrel use (%) | 127 (57.7) | 135 (57.7) | 1.000 | 76 (37.6) | 55 (45.8) | 0.160 |
| Statin use (%) | 172 (78.2) | 174 (74.4) | 0.378 | 132 (65.3) | 80 (66.7) | 0.903 |

Continuous variables described as mean (standard deviation) and categorical variables as count (proportion). Abbreviations: GFR = glomerular filtration rate, CABG = coronary artery bypass grafting, PAD = peripheral artery disease, MI = myocardial infarction, ACEi = angiotensin converting enzyme inhibitor, ARB = angiotensin-II receptor blocker
