# Supplementary material for: Untargeted high-resolution plasma metabolomic profiling predicts outcomes in patients with coronary artery disease
Source: PLoS One. 2020 Aug 18;15(8):e0237579. doi: 10.1371/journal.pone.0237579 (PMC7444579; doi:10.1371/journal.pone.0237579)
Supplement: S3 Table — (DOCX) [file pone.0237579.s007.docx]

**S3 Table: Metabolic pathways associated with all-cause mortality in the first and second cohorts**

| **First Cohort** | | | | **Second Cohort** | | | | |
| --- | --- | --- | --- | --- | --- | --- | --- | --- |
| **Pathway** | **Overlap size** | **Pathway size** | **p-value** | **Pathway** | **Overlap size** | **Pathway size** | | **p-value** |
| Urea cycle/amino group* | 7 | 29 | 0.0009 | Tryptophan* | 5 | 38 | | 0.0029 |
| Glutamate | 3 | 5 | 0.0009 | Urea cycle/amino group* | 4 | 29 | | 0.0039 |
| Aspartate/Asparagine* | 7 | 39 | 0.0023 | Omega-6 fatty acid | 2 | 8 | | 0.0009 |
| Nitrogen | 2 | 3 | 0.0027 | Tyrosine* | 5 | 58 | | 0.0096 |
| Lysine* | 4 | 18 | 0.0032 | Carnitine shuttle* | 3 | 30 | | 0.0176 |
| Butanoate | 4 | 18 | 0.0032 | Vitamin E | 2 | 16 | | 0.0318 |
| Glycosphingolipid | 4 | 22 | 0.0065 | Aspartate/Asparagine* | 3 | 39 | | 0.0394 |
| Alanine/Aspartate | 3 | 12 | 0.0046 | Lysine* | 2 | 18 | | 0.0408 |
| Aminosugars | 3 | 13 | 0.0058 | Fatty acid activation | 2 | 19 | | 0.0458 |
| Arginine/Proline | 3 | 22 | 0.0065 |  |  |  |  | |
| Glycine, Serine, Alanine, and Threonine | 5 | 28 | 0.0045 |  |  |  |  | |
| Tryptophan* | 5 | 38 | 0.0189 |  |  |  |  | |
| Beta-alanine | 2 | 9 | 0.0210 |  |  |  |  | |
| Drug metabolism | 2 | 9 | 0.0210 |  |  |  |  | |
| Tyrosine* | 7 | 58 | 0.0214 |  |  |  |  | |
| Carnitine shuttle* | 4 | 30 | 0.0238 |  |  |  |  | |
| Histidine | 2 | 10 | 0.0268 |  |  |  |  | |
| Pyrimidine | 3 | 21 | 0.0283 |  |  |  |  | |
| Purine | 3 | 21 | 0.0283 |  |  |  |  | |
| Hexose phosphorylation | 2 | 11 | 0.0335 |  |  |  |  | |
| Nicotinate/Nicotinamide | 2 | 12 | 0.0410 |  |  |  |  | |

*Common significant pathways between first and second cohorts
