# Supplementary material for: Untargeted high-resolution plasma metabolomic profiling predicts outcomes in patients with coronary artery disease
Source: PLoS One. 2020 Aug 18;15(8):e0237579. doi: 10.1371/journal.pone.0237579 (PMC7444579; doi:10.1371/journal.pone.0237579)
Supplement: S4 Table — (DOCX) [file pone.0237579.s008.docx]

**S4** **Table: Association of significant metabolites and metabolomic risk score with cardiovascular biomarkers**

|  | **Hs-CRP** | | | **Hs-cTnI** | | | **NT-proBNP** | |
| --- | --- | --- | --- | --- | --- | --- | --- | --- |
|  | **Beta-Coefficient (95% CI)** | **p-value** | | **Beta-Coefficient (95% CI)** | **p-value** | | **Beta-Coefficient (95% CI)** | **p-value** |
| **First Cohort** |  | |  |  | | |  |  |
| m/z 115.0693, rt 55 | 0.40 (-0.35, 1.16) | | 0.297 | 0.49 (0.16, 0.83) | | 0.004 | 1.11 (0.89, 1.33) | <0.001 |
| m/z 188.1755, rt 51 | 0.06 (-0.54, 0.66) | | 0.846 | 0.34 (0.07, 0.61) | | 0.013 | 0.38 (0.19, 0.58) | <0.001 |
| m/z 207.1106, rt 65 | 0.40 (-0.24, 1.04) | | 0.259 | 0.59 (0.31, 0.87) | | <0.001 | 0.72 (0.52, 0.92) | <0.001 |
| m/z 444.6726, rt 55 | 0.13 (-0.55, 0.81) | | 0.715 | -0.28 (-0.58, 0.02) | | 0.073 | -0.30 (-0.52, -0.08) | 0.007 |
| m/z 559.2977, rt 388 | -2.01 (-2.80, -1.21) | | <0.001 | -0.43 (-0.79, -0.07) | | 0.020 | -0.39 (-0.66, -0.13) | 0.003 |
| m/z 1050.6578, rt 412 | -1.02 (-1.70, -0.33) | | 0.003 | -0.23 (-0.53, 0.08) | | 0.151 | -0.26 (-0.49, -0.04) | 0.023 |
| m/z 1078.627, rt 412 | -1.13 (-1.77, -0.49) | | <0.001 | -0.56 (-0.84, -0.27) | | <0.001 | -0.49 (-0.70, 0.29) | <0.001 |
| **Second Cohort** |  | | |  | | |  |  |
| m/z 115.0693, rt 55 | -0.69 (-2.02, 0.65) | | 0.316 | 0.87 (0.52, 1.23) | | <0.001 | 0.95 (0.67, 1.24) | <0.001 |
| m/z 188.1755, rt 51 | 0.22 (-1.13, 1.58) | | 0.748 | 0.18 (-0.18, 3.04) | | 0.344 | 0.24 (-0.05, 0.53) | 0.110 |
| m/z 207.1106, rt 65 | -0.26 (-1.49, 0.97) | | 0.678 | 0.60 (0.28, 0.93) | | <0.001 | 0.56 (0.30, 0.83) | <0.001 |
| m/z 444.6726, rt 55 | 1.40 (0.18, 2.62) | | 0.025 | -0.42 (-0.75, -0.10) | | 0.011 | -0.43 (-0.69, -0.17) | 0.001 |
| m/z 559.2977, rt 388 | -1.29 (-2.66, 0.08) | | 0.067 | -0.67 (-1.04. -0.30) | | <0.001 | -0.46 (-0.75, -0.16) | 0.003 |
| m/z 1050.6578, rt 412 | -0.72 (-1.92, 0.47) | | 0.234 | -0.39 (-0.71, -0.07) | | 0.017 | -0.36 (-0.61, -0.10) | 0.007 |
| m/z 1078.627, rt 412 | -1.06 (0.15, -2.48) | | 0.377 | -0.57 (-0.95, -0.20) | | 0.003 | -0.46 (-0.77, -0.16) | 0.003 |

Linear regression models to determine association of feature intensities (log-transformed) with cardiovascular biomarker levels (log-transformed). Each model adjusted for age, sex, race, and batch effect. Abbreviations: hs-CRP = high-sensitivity C-reactive protein, hs-cTnI = high-sensitivity cardiac troponin-I, NT-proBNP = N-terminal of prohormone brain natriuretic peptide, CI = confidence interval.
