# Supplementary material for: Untargeted high-resolution plasma metabolomic profiling predicts outcomes in patients with coronary artery disease
Source: PLoS One. 2020 Aug 18;15(8):e0237579. doi: 10.1371/journal.pone.0237579 (PMC7444579; doi:10.1371/journal.pone.0237579)
Supplement: S5 Table — (DOCX) [file pone.0237579.s009.docx]

**S5 Table: Association of metabolomic risk score with death after adjustment for cardiovascular biomarkers in the second and combined cohorts**

|  | **Second cohort** | | | **Combined cohort** | |
| --- | --- | --- | --- | --- | --- |
|  | **HR (95% CI)** | **p-value** | | **HR (95% CI)** | **p-value** |
| Per 1-SD increase |  | |  |  | |
| Model* + hs-CRP | 2.14 (1.62, 2.83) | | <0.001 | 1.99 (1.64, 2.40) | <0.001 |
| Model* + hs-cTnI | 1.94 (1.44, 2.61) | | <0.001 | 1.93 (1.59, 2.35) | <0.001 |
| Model* + NT-proBNP | 1.74 (1.29, 2.35) | | <0.001 | 1.59 (1.29, 1.96) | <0.001 |
| Model* + 3 biomarkers | 1.73 (1.27, 2.34) | | <0.001 | 1.59 (1.29, 1.96) | <0.001 |
| Above/Below Median^†^ |  | |  |  |  |
| Model* + hs-CRP | 2.37 (1.25, 4.48) | | 0.007 | 2.29 (1.57, 3.35) | <0.001 |
| Model* + hs-cTnI | 2.28 (1.20, 4.36) | | 0.012 | 2.20 (1.50, 3.23) | <0.001 |
| Model* + NT-proBNP | 2.32 (1.19, 4.52) | | 0.001 | 1.90 (1.28, 2.94) | 0.002 |
| Model* + 3 biomarkers | 2.26 (1.15, 4.43) | | 0.017 | 1.88 (1.26, 2.83) | 0.002 |

* Model adjusted for age (dichotomized at 75 years), current smoking, hypertension, diabetes, HF, PAD, stroke, prior CABG, eGFR (dichotomized at 60 ml/min/1.73 m^2^), and batch effect. ^†^Individuals with lower than median metabolomic risk score are the reference group.

Biomarker levels were log-transformed, and hs-CRP was not associated with mortality in second (HR 1.01, 95% CI 0.98-1.04, p=0.586) and combined (HR 1.04, 95% CI 0.99-1.08, p=0.073) cohorts. However, hs-cTnI (first HR 1.14, 95% CI 1.06-1.22, p<0.001; and second HR 1.11, 95% CI 1.07-1.16, p<0.001) and NT-proBNP (first HR 1.43, 95% CI 1.28-1.61, p<0.001; and second HR 1.43, 95% CI 1.34-1.54, p<0.001) were associated with mortality. Abbreviations: HF =heart failure, PAD = peripheral artery disease, CABG = coronary artery bypass grafting, eGFR = estimated glomerular filtration rate.
