# Supplementary material for: Untargeted high-resolution plasma metabolomic profiling predicts outcomes in patients with coronary artery disease
Source: PLoS One. 2020 Aug 18;15(8):e0237579. doi: 10.1371/journal.pone.0237579 (PMC7444579; doi:10.1371/journal.pone.0237579)
Supplement: S6 Table — (DOCX) [file pone.0237579.s010.docx]

**S6 Table: Association of metabolomic risk score with cardiovascular death in the second and combined cohorts**

|  | **Second cohort** | | | **Combined cohort** | | |
| --- | --- | --- | --- | --- | --- | --- |
|  | **HR (95% CI)** | **p-value** | | **HR (95% CI)** | **p-value** | |
| Per 1-SD increase |  | |  |  | | |
| Unadjusted | 2.32 (1.52, 3.54) | | <0.001 | 2.09 (1.63, 2.69) | | <0.001 |
| Model 1* | 2.60 (1.66, 4.08) | | <0.001 | 2.07 (1.60, 2.67) | | <0.001 |
| Above/Below Median^†^ |  | | |  | | |
| Unadjusted | 3.40 (1.30, 8.88) | | 0.012 | 2.79 (1.65, 4.73) | | <0.001 |
| Model 1* | 3.44 (1.27, 9.34) | | 0.015 | 2.63 (1.55, 4.47) | | <0.001 |

* Model 1 adjusted for age, sex, race, and batch effect. ^†^Individuals with lower than median metabolomic risk score are the reference group. Abbreviations: MI = myocardial infarction, HR = hazard ratio, CI = confidence interval, and SD = standard deviation.
