# Supplementary material for: Untargeted high-resolution plasma metabolomic profiling predicts outcomes in patients with coronary artery disease
Source: PLoS One. 2020 Aug 18;15(8):e0237579. doi: 10.1371/journal.pone.0237579 (PMC7444579; doi:10.1371/journal.pone.0237579)
Supplement: S7 Table — (DOCX) [file pone.0237579.s011.docx]

**S7 Table: Improvement in cardiovascular death risk discrimination and risk reclassification indices with metabolomic risk score***

|  | **Second cohort** | | **Combined cohort** | |
| --- | --- | --- | --- | --- |
|  | **Estimate (95% CI)** | **p-value** | **Estimate (95% CI)** | **p-value** |
| Baseline C-statistic | 0.649 (0.512, 0.785) | - | 0.570 (0.476, 0.665) | - |
| Delta C-statistic | 0.100 (-0.029, 0.229) | - | 0.113 (0.026, 0.201) | - |
| IDI | 0.077 (0.012, 0.190) | 0.002 | 0.048 (0.020, 0.100) | <0.001 |
| NRI | 30.5% (5.6%, 49.7%) | 0.014 | 28.5% (15.7%, 40.7%) | <0.001 |

*Baseline model consists of age, sex, and race. Abbreviations: MI = myocardial infarction, CI = confidence interval, IDI = Integrated Discrimination Index, and NRI = Net Reclassification Index.
